# 

**Published:** 1864-07

**Authors:** 


### CHICAGO MEDICAL JOURNAL.

**Terms, $2.00 per Annum.**

### CONTENTS OF THE JULY NUMBER.

PAGE.

ORIGINAL COMMUNICATIONS.

Report of Special Committee on Diseases of the Eye, By E. L. Holmes, M. D., of Chicago, Lecturer, etc. .......... 298

Rambles in Military Hospitals. Report of a Case of Encysted Abscess of the Brain, By R. M. Lackey, M. D., A. A. Surg. U. S. A.. .......... 300

A Case of Metropolypus, By D. B. Trimble, M. D .......... 303

Removal of a Fibro-Cellular Tumor of the Scrotum, By A. J. Baxter, M. D., of Chicago .......... 309

Paper on Cerebro-Spinal Meningitis. Read before the Illinois State Med. Soc. at its annual Meeting in May, 1864, By R. E. McVey, M. D., Waverly, Ill .......... 310

Cerebro-Spinal Meningitis, By M. M. Eaton, M D., of Peoria, Ill .......... 312

SELECTED.

Code of Medical Ethics .......... 315

EDITORIAL AND MISCELLANEOUS .......... 329

BOOK NOTICES AND REVIEWS .......... 335

